# Home oxygen monitoring and therapy: learning from the pandemic

**DOI:** 10.1097/MCC.0000000000001010

**Published:** 2022-12-20

**Authors:** Thomas Beaney, Jonathan Clarke

**Affiliations:** a Department of Primary Care and Public Health; b Department of Mathematics, Imperial College London, London, UK

**Keywords:** oxygen monitoring, oxygen therapy, remote monitoring, telehealth, telemonitoring

## Abstract

Home oxygen monitoring and therapy have been increasingly used in the management of patients with chronic diseases. The COVID-19 pandemic has prompted the rapid uptake of remote monitoring programmes to support people with COVID-19 at home. This review discusses the recent evidence and learning in home oxygen monitoring and therapy from the pandemic.

**Recent findings:**

Many home oxygen monitoring programmes were established around the world during the pandemic, mostly in high-income countries to support early detection of hypoxaemia and/or early hospital discharge. The characteristics of these programmes vary widely in the type of monitoring (self-monitoring or clinician-monitoring) and the patient risk groups targeted. There is a lack of evidence for benefits on clinical outcomes, including mortality, and on reductions in healthcare utilisation or cost-effectiveness, but programmes are viewed positively by patients. Recent studies have highlighted the potential bias in pulse oximetry in people with darker skin.

**Summary:**

Recent evidence indicates that home oxygen monitoring therapy programmes are feasible in acute disease, but further research is needed to establish whether they improve patient outcomes, are cost-effective and to understand their equity impact.

## INTRODUCTION

The last two decades have seen the growth of telemedicine and remote monitoring to enable healthcare delivery to patients at home [1,2]. The threat to global health systems resulting from the COVID-19 pandemic has accelerated the adoption of new ways of working across the globe and an increased use of remote monitoring, including oxygen monitoring and use of home oxygen therapy.

The current review provides an update on learning from the development of remote oxygen monitoring and therapy programmes during the COVID-19 pandemic, covering the scope and purpose of programmes, type of monitoring and targeted populations. We also discuss the evidence-base for the clinical effectiveness and cost-effectiveness of monitoring and therapy, and, finally, provide an overview of the evidence for potential inequalities that may arise from remote oxygen monitoring. 

**Box 1:**
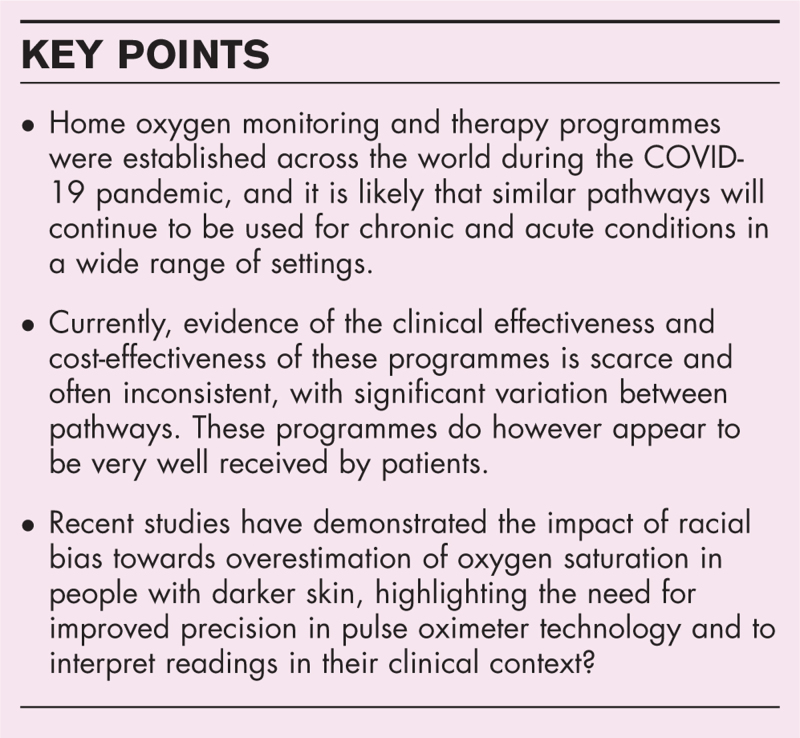
no caption available

## BACKGROUND

Remote oxygen monitoring technologies have been used for that long?, particularly for chronic diseases such as chronic obstructive pulmonary disease (COPD) and heart failure, although evidence of benefits of these programmes is unclear [2,3]. The COVID-19 pandemic has led to the rapid establishment of remote oxygen saturation monitoring programmes around the world. Early in the pandemic, low oxygen saturations were identified as a key predictor of mortality and the need for hospital admission [4], but low oxygen saturations in the absence of symptoms (so-called silent hypoxaemia) was recognized as a feature of many COVID-19 infections [5]. Pulse oximetry was thus identified as a route to early detection of the need for further clinical assessment and escalation of care and has led to the widespread adoption of monitoring programmes [6].

Oxygen therapy at home has been used for decades in the management of patients with severe hypoxaemia in COPD following landmark trials in the 1980s with a strong evidence base for increased survival [7,8]. However, its use is not limited to COPD, with the British Thoracic Society guidelines on the use of home oxygen in adults, for example, recommending its use in a selection of other chronic respiratory or cardiac diseases and include its use in long-term, nocturnal, ambulatory, palliative and ‘short-burst’ contexts, with differing recommendations for the prescription and monitoring in each case [9]. However, there is little evidence prior to the pandemic for home oxygen therapy in the management of acute diseases. A recent meta-analysis examining the use of home oxygen for moderate hypoxaemia in COPD found little or no difference in 3-year mortality as a result of home oxygen therapy [10]. The recent review of the American Thoracic Society guidelines acknowledge that ‘available data on home oxygen therapy are limited and generally of low quality and most directly support the recommendations pertaining to patients with severe room air hypoxemia due to COPD.’ [11] Despite this, home oxygen therapy has been used in several settings as part of the treatment of acute COVID-19 disease.

## PURPOSE AND SCOPE OF HOME OXYGEN MONITORING AND THERAPY PROGRAMMES

Since early 2020, many home monitoring programmes which include oxygen saturation monitoring have been described for patients with COVID-19, predominantly from high-income countries, including studies from Australia [12], Belgium [13], Canada [14], Germany [15], Japan [16], the Netherlands [17], South Africa [18], United Kingdom [19–23] and the USA [24–27]. The majority of models have served one or both of two main goals: first, early detection of hypoxaemia to escalate care for people in the community with COVID-19 [14–16] or second, to enable early discharge from hospital in patients with COVID-19 [13,19,24,25]. Oxygen monitoring programmes have also been used to support clinical assessment and triage, rather than ongoing monitoring, for example a UK programme delivering equipment to patients to record observations, including a pulse oximeter and digital stethoscope, to enable clinicians to assess the need for hospital assessment or admission remotely [28].

A systematic review of home oxygen monitoring programmes implemented during the COVID-19 pandemic found significant variation in the characteristics of programmes, with variation in the frequency of monitoring, mode of submitting oxygen saturation measurements and total follow-up time [29^▪^]. Many of the oxygen monitoring programmes also existed as part of a wider package of remote monitoring, for example, including thermometers and blood pressure devices or including additional symptom monitoring [29^▪^].

Supplemental oxygen therapy has been described as part of relatively few remote monitoring pathways, used either to prevent the need for admission to hospital or to facilitate early supported discharge from hospital [16,30–32]. Some pathways were designed solely for those requiring home oxygen therapy, whereas others offered home oxygen therapy as part of a wider oximetry monitoring programme. These findings highlight that there is no single model of monitoring and therapy delivery and that programmes should be tailored to the specific population and setting.

## SELF-MONITORING OR CLINICIAN MONITORING?

Home oxygen monitoring programmes vary in the intensity of staff input, and in whether patients self-monitor and self-escalate, are prompted to record readings, or are contacted and assessed proactively by clinical staff. A programme in Scotland, for example, which provided pulse oximeters to patients with COVID-19 at home, took the form of self-monitoring, with patients reminded twice daily to record symptoms and oxygen saturation readings, via SMS, app or online, receiving advice on escalation depending on symptoms and oxygen saturations [23]. In contrast, a study reporting on programmes implemented across England highlighted the role of clinician discretion in the modality and frequency of monitoring calls from programme staff. In some sites, patients could input measurements onto an app, which was reviewed by clinicians, but also allowed for automated alerts to patients, such as prompting a call to emergency services [33^▪^]. A monitoring and therapy programme in Japan involved a much more intensive ‘hospital-at-home’ service, including daily nurse and physician visits for higher risk patients [16].

## WHO SHOULD BE MONITORED?

In the context of COVID-19, while some monitoring programmes have invited all patients with COVID-19 [14,15], most programmes have catered for higher risk patients. A programme in Japan focussed on people aged 70 years or over, but also included people with specific higher risk criteria (e.g., mental illness or with limited proficiency in Japanese) [16]. A national monitoring programme in England was initially recommended for patients aged 65 years or over, or at high-risk from COVID-19, but criteria were later extended to those aged 50 years or over [34]. The high-risk criteria included particular comorbidities, but was also based on the QCovid risk calculator, which includes factors such as ethnicity and measures of socioeconomic deprivation [34,35]. The programme also allowed for clinical discretion in decisions to enrol patients, in keeping with a programme in the USA, which gave full discretion to clinicians enrol patients [24]. Other bespoke programmes have been reported for specific demographic groups, such as those for pregnant women with confirmed COVID-19 in England, the Netherlands and the USA [17,36,37].

## EVIDENCE FOR THE EFFECTIVENESS OF REMOTE OXYGEN MONITORING

Prior to the pandemic, evidence for the effectiveness of remote oxygen monitoring in improving clinical outcomes and reducing healthcare use in chronic diseases was unclear. A recent systematic review of studies reporting on remote patient monitoring identified that it may reduce acute care use in people with cardiovascular disease and COPD, but identified significant heterogeneity between studies, which prevented meta-analysis [38].

Since the start of the COVID-19 pandemic, several studies have reported on safety, healthcare utilization and clinical outcomes in patients on monitoring programmes. A study of four monitoring sites in England early in the pandemic showed that of 291 patients who were at low risk (<65 years and with no comorbidities), there were no deaths, with the authors concluding that home oxygen monitoring was safe [39].

Several observational studies have reported associations with clinical outcomes for those enrolled onto monitoring programmes compared with control groups. A study in the USA found patients discharged with remote monitoring (provided with a pulse oximeter and thermometer) had 46% lower odds (*P* = 0.039) of emergency department (ED) or hospital readmission compared with those discharged without monitoring [40]. A single-site study in England found that admitted patients who had previously been enrolled on a monitoring programme had significantly lower 30-day mortality and hospital length of stay, but found no difference in intensive care admissions to patients admitted directly without having been enrolled on a monitoring programme, after adjusting for age, sex and comorbidities [20]. However, in keeping with several other single-site observational studies, enrolment to these programmes was not randomized; selective enrolment of patients with less severe disease or at different stages of disease onset may introduce significant bias to estimates.

Other studies have attempted to mitigate the impact of confounding by patient selection using population-based or instrumental variable designs. Two separate evaluations of a national oxygen monitoring programme in England using these approaches found no impact of the programme on mortality and no clear association with subsequent healthcare attendances [34,41]. However, both studies acknowledged small numbers enrolled onto the programme, which may have underpowered the studies to detect any effect and with findings likely reflecting background trends in population risk rather than of home monitoring.

A randomized-controlled trial (RCT) published in 2022 randomized 1041 patients to standard home monitoring, involving twice daily messages asking about dyspnoea, versus 1056 to an intervention arm providing a pulse oximeter, and found no significant differences in the number of days alive and out of hospital between groups [42^▪^]. However, the control arm of this study involved a degree of clinician-initiated review, which is unlikely to be standard care in most healthcare settings and may lead to underestimation of any impact of pulse oximetry monitoring.

The evidence for the effectiveness of monitoring programmes on mortality and healthcare utilization remains unclear, with a need for further RCTs or observational studies applying causal inference methods. However, interpretation may be further impacted by the significant heterogeneity already highlighted between how programmes are implemented.

## EVIDENCE FOR THE EFFECTIVENESS OF OXYGEN THERAPY

In a cohort of 621 patients with COVID-19 pneumonia in the USA discharged from hospital with home oxygen, rates of readmission (8.5%) and all-cause mortality (1.3%) at 28 days were low. No deaths occurred in the ambulatory setting, however, the absence of a control group in this study means findings should be interpreted with caution [31]. A similar study from the United Kingdom of patients with COVID-19 monitored at home with pulse oximetry found no significant difference in the odds of admission or mortality within 30 days of discharge from hospital between those receiving and those not receiving home oxygen [32]. A study from the Netherlands of 196 patients receiving early supported hospital discharge onto a home oxygen programme for the treatment of COVID-9 was associated with a reduction in length of hospital stay of 6.4 days [30].

Although evidence for the benefit of home oxygen therapy for the treatment of COVID-19 is scarce, its use in clinical pathways in several countries and clinical settings suggests it is both feasible and potentially clinically useful to incorporate the provision of home oxygen as a supplement to home monitoring pathways for those where it may be clinically indicated.

## COST-EFFECTIVENESS

Some studies have demonstrated that remote monitoring programmes may be cost effective. A cost-effectiveness analysis of patients presenting to EDs with moderate-to-severe COVID-19 found that those discharged from ED on a remote oximetry pathway had lower rates of mortality, subsequent ED visits and intensive care admissions in the 3 weeks following presentation than those not on the pathway, amounting to an estimated saving of $11 472 per patient [43]. Importantly, this analysis was unable to account for the other clinical characteristics of those who did and did not receive the intervention. Beyond this study, formal estimation of the cost-effectiveness of remote monitoring and remote therapy programmes in COVID-19 is limited by a lack of consistent findings of clinical effectiveness.

Aside from formal cost-effectiveness analyses, survey findings may also support the potential cost-effectiveness of remote monitoring programmes. In a remote monitoring pathway initiated following ED attendance, 33% of those enrolled stated they would have returned to the ED if they did not have reassurance provided by the pulse oximeter at home [44]. In a cohort of oncology patients with COVID-19, 59% agreed that participation in a remote monitoring programme helped prevent ED or urgent care centre visits [45]. Taken collectively, there is promise that such pathways may offer a cost-effective way to provide care, however most of the evidence for effectiveness is demonstrated in the preferences of patients and clinicians, rather than through consistently demonstrated differences in clinical outcomes and health services utilization.

## FEASIBILITY AND ACCEPTABILITY

Despite variable and scarce evidence of the clinical and cost effectiveness of home oximetry programmes, a largely consistent finding across studies is the positive reception of such programmes by patients. In a study of 4400 patients enrolled in a COVID-19 home monitoring programme in the USA, around 78% of participants were satisfied with the programme and 97% of respondents felt safe monitoring their condition at home [46]. In a cohort of 257 oncology patients diagnosed with COVID-19 who were enrolled onto a pulse oximetry remote monitoring programme, 91% felt participation in the programme was helpful, 87% felt that participation was an important part of their care and 76% felt participation helped manage their symptoms at home [45]. In a Dutch study combining remote monitoring and home oxygen administration, 98% of patients would recommend the service while 93% rated it as ‘user friendly’ [30].

In a multisite survey of COVID-19 remote home monitoring models in England, patient experience was described by staff as generally positive, with reassurance being a major advantage of the programmes [33^▪^]. They did, however, also report some patients becoming more anxious as a result of monitoring and a reduction in patient engagement with monitoring was observed in the latter stages of the pandemic.

## DISPARITIES IN HOME MONITORING AND THERAPY

As with any innovation in healthcare, the benefits of home oximetry monitoring and oxygen therapy may be experienced differently across patient groups [47^▪^]. Although several studies show patients found remote monitoring services easy to use, concerns remain for those with lower digital literacy or with cognitive or physical conditions that would make recording and responding to information difficult [48]. Similarly, remote monitoring pathways often rely upon an established clinical information sharing infrastructure, usually in the form of electronic health records, supplemented by app-based interfaces [49,50]. Such infrastructure may not exist, or only be available to a small proportion of the population, particularly in lower income countries [49,50]. As such, there is a risk that such pathways may widen rather than narrow inequities in access to clinical services. Designing and funding services with these considerations in mind is crucial to their fair and successful implementation.

A separate concern, first identified over 20 years ago, which came to prominence during the COVID-19 pandemic relates to the effectiveness of pulse oximeters in identifying hypoxaemia in those with darker skin [51]. Two recent studies find pulse oximeters fail to identify hypoxaemia more frequently in patients with darker skin, who as a result may be assessed as having less severe disease than White patients with identical arterial oxygen saturations and may thus be less likely to receive treatment [52,53^▪▪^]. Both studies also demonstrate extensive disagreement between oxygen saturation levels estimated by pulse oximetry and arterial blood gas analysis. Collectively, this indicates a need to both improve the precision of pulse oximeters overall, and specifically for patients with darker skin, and highlights the need for measurements to be interpreted in their clinical context.

## CONCLUSION

Remote oxygen monitoring has been used for many years for a range of mostly chronic conditions, however pressures from the COVID-19 pandemic have seen a dramatic expansion in the scale of such models of care. A wide range of programmes have been established, some focussed on early detection of hypoxaemia and others to enable early discharge, and with heterogeneity in the mode and intensity of monitoring and targeted population. Evidence for the effectiveness of home oxygen monitoring and oxygen therapy on patient outcomes in the context of COVID-19 is unclear, but programmes appear to be well received by patients and clinicians alike. However, there remain significant concerns about the equity impact of pulse oximeters, particularly in people with darker skin. Rapid increases in the maturity and sophistication of digital clinical devices have made remote care a feasible alternative to face-to-face care. Such pathways are likely to persist and expand in the coming years to eventually become the norm for those whose clinical needs can be met without the need to attend a hospital or clinic.

## Acknowledgements

*None.*

### Financial support and sponsorship

*T.B. and J.C. are supported by fellowships from the Wellcome Trust. The views expressed in this publication are those of the authors and not necessarily those of their institutions or the Wellcome Trust.*

### Conflicts of interest

*The authors report no conflicting interests.*

## REFERENCES AND RECOMMENDED READING

Papers of particular interest, published within the annual period of review, have been highlighted as:

▪ of special interest
▪▪ of outstanding interest
